# Editorial: The digitalization of neurology

**DOI:** 10.3389/fdgth.2023.1291110

**Published:** 2023-10-09

**Authors:** Daniel B. Hier, Michael D. Carrithers, Jorge M. Rodríguez-Fernández, Benjamin Kummer

**Affiliations:** ^1^ Department of Neurology and Rehabilitation, University of Illinois at Chicago, Chicago, IL, United States; ^2^ Kummer Institute for Artificial Intelligence and Autonomous Systems, Missouri University of Science and Technology, Rolla, MO, United States; ^3^ Department of Neurology, University of Texas Medical Branch, Galveston, TX, United States; ^4^ Department of Neurology, Icahn School of Medicine at Mount Sinai, New York, NY, United States

**Keywords:** neurology, digitalization, precision medicine, precision neurology, teleneurology, computation

**Editorial on the Research Topic** The digitalization of neurology

## Introduction

Modern neurology originated in the nineteenth century with the writings of Charcot, Wernicke, Gowers, Hughlings Jackson, Cajal, Broca, and others (1). For much of the history of neurology, observations were qualitative, not quantitative. Case reports and small case series dominated disease descriptions. Neuroimaging was characterized by plain radiographs that were not suitable for computer analysis. EEG and EMG waveforms were analog and not digital. Medical records were on paper and were not suitable for computer analysis. Patients were examined in person. Before modern neuroimaging, a definitive diagnosis was often impossible without a biopsy or autopsy.

*We define the digitalization of neurology as the transition to computable observations, treatments, diagnoses, and outcomes.*

Neurology digitalization began slowly 50 years ago and has accelerated in the last 20 years. Progress has occurred on multiple fronts, including *Precision Neurology, Big Data in Neurology, Computable Neurology*, and *Remote Neurology*.

## Precision neurology

*Precision neurology is the application of precision medicine principles to the field of neurology.*

Precision neurology involves tailoring neurological care and treatment to individual patients according to their unique genetic, molecular, and clinical profiles. In this Research Topic, *Differential DNA methylation associated with multiple sclerosis and disease modifying treatments in an underrepresented minority population* by Bingen et al. found that MS is associated with differential DNA methylation in genes that regulate immune cell differentiation, host defense against gastrointestinal pathogens, and susceptibility to acute myeloid leukemia. They also found an epigenetic signature associated with dimethyl fumarate treatment in genes that regulate cytokine signaling, axon guidance, and adherens junctions.

In another example of precision neurology, *Designing evidence-based support aids for social media access for individuals with moderate-severe traumatic brain injury: A preliminary acceptability study* by Zhao et al. examines the feasibility of modifying access to social networks for people affected by brain injury.

Another precision neurology study by Howlett-Prieto et al. is *Subtypes of relapsing-remitting multiple sclerosis identified by network analysis*. They used network analysis of multiple sclerosis phenotypes to identify the first subtypes of relapsing and remitting multiple sclerosis that could differ in response to treatment or outcome.

## Big data in neurology

*Big data in neurology is the creation of large data sets that have both depth (many patients) and breadth (many variables that include data from imaging, proteomics, clinical observations, genomics, etc.)*

In a big data approach to epilepsy, *INTUITION: a data platform to integrate human epilepsy clinical care and support for discovery* by Maharathi et al. describes the merging of pathological, clinical, radiographic, pharmacological and electroencephalographic data captured in the surgical treatment of epilepsy.

Another article from this Research Topic *Parkinson’s disease population-wide registries in the United States: Current and future opportunities* examines opportunities and barriers to the creation of large Parkinson’s disease registries in the United States (Wu and Wilson). The California Parkinson’s Disease Registry has already collected information on 93,928 unique Parkinson’s disease patients.

Another article from this Research Topic is entitled *Workflow for health-related and brain data lifecycle* and it examines best practices for curating and maintaining data related to brain health (Brůha et al.).

## Computable neurology

*Computable neurology converts neurological observations into machine-readable codes that can be entered into machine learning and deep learning applications.*

Traditional observations in neurology have been qualitative rather than quantitative. Computation with qualitative observations has been difficult. Initially, electroencephalographic and electromyographic waveforms were analog. The digitalization of these waveforms has made them computable. Similarly, analog images on radiographic films have been made computable by digitalization.

The Hecker et al. article *Voice Analysis for Neurological Disorder Recognition–A Systematic Review and Perspective on Emerging Trends* illustrates how voice features can be digitally encoded to enhance the diagnosis and treatment of neurological disorders.

Medical records on paper have been converted to electronic health records. However, unstructured patient data in electronic health records must be normalized using ontologies and natural language processing methods to create computable concepts suitable for machine learning and deep learning applications. Several articles examine methods for extracting computable concepts from electronic health records, including methods for annotating neurological concepts. Azizi et al.’s *Enhanced neurologic concept recognition using a named entity recognition model based on transformers* explores the use of neural networks to extract neurological concepts from unstructured text. *Inter-rater agreement for the annotation of neurologic signs and symptoms in electronic health records* by Oommen et al. evaluates how well different raters perform in identifying neurological concepts in free text from electronic health records. After phenotypes have been extracted from electronic health records, *The visualization of Orphadata neurology phenotypes* explores the visualization of these neurological phenotypes with heat maps and word clouds (Hier et al.).

Although extracting concepts from unstructured text in electronic health records shows great promise for precision neurology and big data, documentation has burdened physicians and other providers. It contributes to physician burnout (2). An article in this Research Topic entitled *It’s time to change our documentation philosophy: writing better neurology notes without the burnout* describes strategies that can reduce documentation burden and take advantage of recent changes in documentation regulations from the Center for Medicare and Medicaid Services (CMS) (Rodríguez-Fernández et al.).

## Remote neurology

*Remote neurology is using technology to provide neurological services at a distance.*

Teleneurology has been used for acute and non-acute neurological consultations, including stroke (3). Another example of remote neurology is monitoring neurological patients by actimetry or telemetry (4, 5). In this Research Topic, Ward et al.’s *Implementation and impact of a point of care electroencephalography platform in a community hospital: a cohort study* demonstrates the feasibility of providing emergency EEG services in a community hospital when neurologists and technicians are not available via a point-of-care EEG device.

The twelve articles in this Research Topic highlight the accelerated pace of digitalization in neurology and illustrate the varied uses of neurological observations once they have been made computable. We believe that further advances in neurology will depend on the increasing digitalization of neurology and that the abundant availability of observations in a computable form will support the implementation of advanced methods from machine learning and artificial intelligence.
